# Commensal microbiome and gastrointestinal mucosal immunity: Harmony and conflict with our closest neighbor

**DOI:** 10.1002/iid3.1316

**Published:** 2024-07-18

**Authors:** Kexin Tian, Dehong Jing, Junzhe Lan, Mingming Lv, Tingting Wang

**Affiliations:** ^1^ The State Key Laboratory of Pharmaceutical Biotechnology, Division of Immunology, Medical School Nanjing University Nanjing China; ^2^ Jiangsu Key Laboratory of Molecular Medicine, Division of Immunology, Medical School Nanjing University Nanjing China; ^3^ Department of Breast Women's Hospital of Nanjing Medical University, Nanjing Maternity, and Child Health Care Hospital Nanjing China

**Keywords:** commensal microbiome, CRC, IBD, intestinal mucosal immunity, mucosal vaccine, oral tolerance

## Abstract

**Background:**

The gastrointestinal tract contains a wide range of microorganisms that have evolved alongside the immune system of the host. The intestinal mucosa maintains balance within the intestines by utilizing the mucosal immune system, which is controlled by the complex gut mucosal immune network.

**Objective:**

This review aims to comprehensively introduce current knowledge of the gut mucosal immune system, focusing on its interaction with commensal bacteria.

**Results:**

The gut mucosal immune network includes gut‐associated lymphoid tissue, mucosal immune cells, cytokines, and chemokines. The connection between microbiota and the immune system occurs through the engagement of bacterial components with pattern recognition receptors found in the intestinal epithelium and antigen‐presenting cells. This interaction leads to the activation of both innate and adaptive immune responses. The interaction between the microbial community and the host is vital for maintaining the balance and health of the host's mucosal system.

**Conclusion:**

The gut mucosal immune network maintains a delicate equilibrium between active immunity, which defends against infections and damaging non‐self antigens, and immunological tolerance, which allows for the presence of commensal microbiota and dietary antigens. This balance is crucial for the maintenance of intestinal health and homeostasis. Disturbance of gut homeostasis leads to enduring or severe gastrointestinal ailments, such as colorectal cancer and inflammatory bowel disease. Utilizing these factors can aid in the development of cutting‐edge mucosal vaccines that have the ability to elicit strong protective immune responses at the primary sites of pathogen invasion.

## Graphical abstract

The intestinal mucosal immune system, intricately linked with commensal microorganisms, orchestrates immune responses to pathogens, and the symbiotic flora plays a crucial role in maintaining a dynamic balance of mucosal immunity. This paper provides a comprehensive overview of the interplay between the two, serving as a valuable resource for future research on their relationships and potential implications for intestinal disorders.

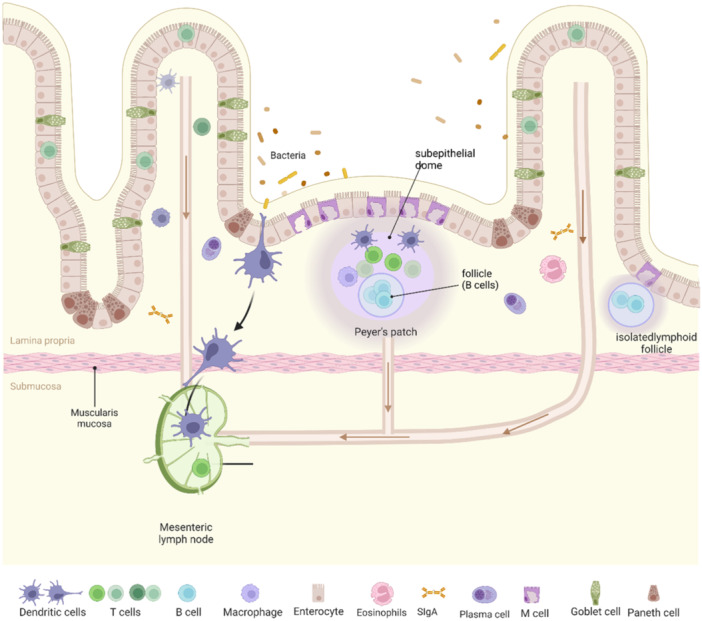

## 1 INTRODUCTION

Mucosal immunity refers to the local immunity of the mucosal tissues and certain glands of the respiratory, genitourinary, and gastrointestinal tracts, which are in contact with the outside world. Its primary function is to remove pathogenic microorganisms that invade the body through the mucosal surface. The mucosal immune system operates separately from the broader immune system but is inseparable from the systemic immune system. The gastrointestinal mucosal system may have been the gateway of direct contact with external antigens, possibly linked to the need to deal with the complex and dynamic populations of commensal bacteria.

The GI tract contains a variety of bacteria, archaea, and eukaryotes, collectively termed the “gut commensal microbiome.” Commensals has formed a relationship of mutually beneficial coexistence with the host over thousands of years. The microbiota that live in the GI tract help the host in many ways such as metabolic function,^1^ trophic function,^2^ immunologic function,^3^ and intestinal defense function.^2^ However, host and gut commensal microbiome's homeostasis can be disrupted due to an altered microbial composition, causing intestinal and extra‐intestinal diseases.

This review outlines our present knowledge of the human GI commensal microbiota and gastrointestinal mucosal immunity, a representative overview of how they interact and impact host health.

## 2 COMMENSAL MICROBIOTA: OUR LONG‐STANDING NEIGHBOR

Vertebrates and bacteria have co‐evolved in intimate contact for more than 150 million years. Over 10^14 microorganisms have been found in the GI tract, which is roughly 10 times more bacterial cells than there are human cells.^4^ Our mucosal surfaces, therefore, involve an integration with the resident microbiota to create a “supraorganism,” referring to the host, and all the microbes living inside it.

Most microbiome studies have focused on bacteria, and less attention has been paid to viruses, fungi, and archaea. However, these rare microbial components have a relatively rich composition. Fungi in the GI are highly susceptible to environmental factors, especially diet.^5^ The three groups that were found the most frequently were mucoromycetes (62.94%–97.56%), ascomycetes (0.89%–31.06%), and basidiomycetes (1.30%–7.63%). Euryarchaeota was the most abundant phylum for archaea, and Methanobacteriaceae was the most common family.^6^ Viruses are lower in content than bacteria but are a stable part of the entire microbial ecosystem, among which Bacteriophages are most identified.^7^

### 2.1 Biogeography of commensal microbiota

Each mucosal surface has cell types and breeds different microenvironments, which result in the biogeography of microbiota. Variation of microbial habitats along the lengths of the GI tract is affected by gradients in nutrients and chemicals as well as segregated host immune activity. For instance, compared to the colon, the small intestine is more acidic, has higher quantities of oxygen, and contains more antimicrobials. Therefore, the microbiome of the small intestine is dominated by facultative anaerobes with rapid growth.^8^

These communities of bacteria evolve with the host and are highly specialized in occupying different niches. From a population of 10^2‐10^3 aerobic organisms/gram luminal contents in the proximal stomach and duodenum to a population of 10^11‐10^12 predominantly anaerobic bacteria/gram in the cecum and colon, these microorganisms increase in both concentration and complexity as they migrate through the digestive tract, according to a molecular characterization of the microbial makeup of fecal and mucosal samples using 16 s ribosomal DNA and RNA. The four classes of bacteria known as Firmicutes, Bacteroidetes, Proteobacteria, and Actinobacteria account for more than 99% of the gut microbiota.^9^, ^10^ The Clostridium XIV and IV groups, which make up the majority of connected colonic species (64%), and the Bacteroidetes, which make up 23% of normal species, are the leading Firmicutes. The Enterobacteriaceae family, which includes *Escherichia coli*, makes up less than 10% of all symbiotic bacteria.^10^ Because of sampling difficulties, studies about small intestinal microbiota are rare. Streptococceae, Lactobacillales, Actinomycinae, and Corynebacteriaeceae (Bacillus subgroup of Firmicutes) were enriched, while Clostridia and Bacteroides were depleted, according to a molecular study of bacteria found in the mucosa^10^ (Table 1).

**Table 1:** Microbial colonization of the GI tract.

Stomach	0–10^2 (CFU/mL)	Lactobacillus
		Candida
		Streptococcus
		Helicobacter pylori
		Peptostragtococcus
		Candida (fungi)
		Phialemonium (fungi)
Duodenum	10–10^3 (CFU/mL)	Streptococcus
Jejunum/lleum	10^4–10^7 (CFU/mL)	Streptococcus
Distal ileum	10^7–10^8 (CFU/mL)	Clostridium
		Bacteroides sp
		Conlidorms
		Methanobrevibacter smithii (archaea)
Colon	10^11–010^12 (CFU/mL)	Bacteroides
		Bifidobacterium
		Clostridium coccoides
		Clostridium leplum
		Fusobacterium
		Ascomycota (fungi)
		Basidiomycota phyla (fungi)

### 2.2 Role of commensal microbiota in health

Additionally to the function of the gut flora in immunity, which we will focus on next, they have a non‐negligible impact on human health in other ways.

To participate in various metabolic processes in the human body, our microbiome joins forces with the host to form a host‐microbiota co‐metabolism structure. Complex carbohydrates can be fermented by bacteria to produce metabolites like SCFAs, which are essential chemical messengers between the microbiota and host. It is hypothesized that SCFA has effects that work together to enhance intestinal, hepatic, and overall glucose homeostasis.^11^ Microbiota metabolism also involves bile acid metabolism,^12^ choline metabolism,^13^ tryptophan metabolism,^14^ etc.

The trophic function of the microbiota cannot be ignored either. By boosting the expression of intestinal nutrient transporters, the gut microbiota supplies nutrients to the host. SCFAs further promote the intestinal gluconeogenesis process to form supporting lipids.^15^ The gastrointestinal microbiota is also involved in the de novo synthesis of essential vitamins that cannot be produced by the host, such as Vitamin B12.^16^ Of particular note is that butyrate is a vital energy source for colonocytes.^17^

## 3 MUCOSAL IMMUNITY: THE FENCE WALL IN BETWEEN

Different inductive and effector sites make up the immune system of the gut mucosa. The inductive sites are the mesenteric lymph nodes, which have the physically compartmentalized organization typical of the peripheral lymphoid organ, and the gut‐associated lymphoid tissues (GALT).^18^, ^19^

**BOX**

**Figure 1:**
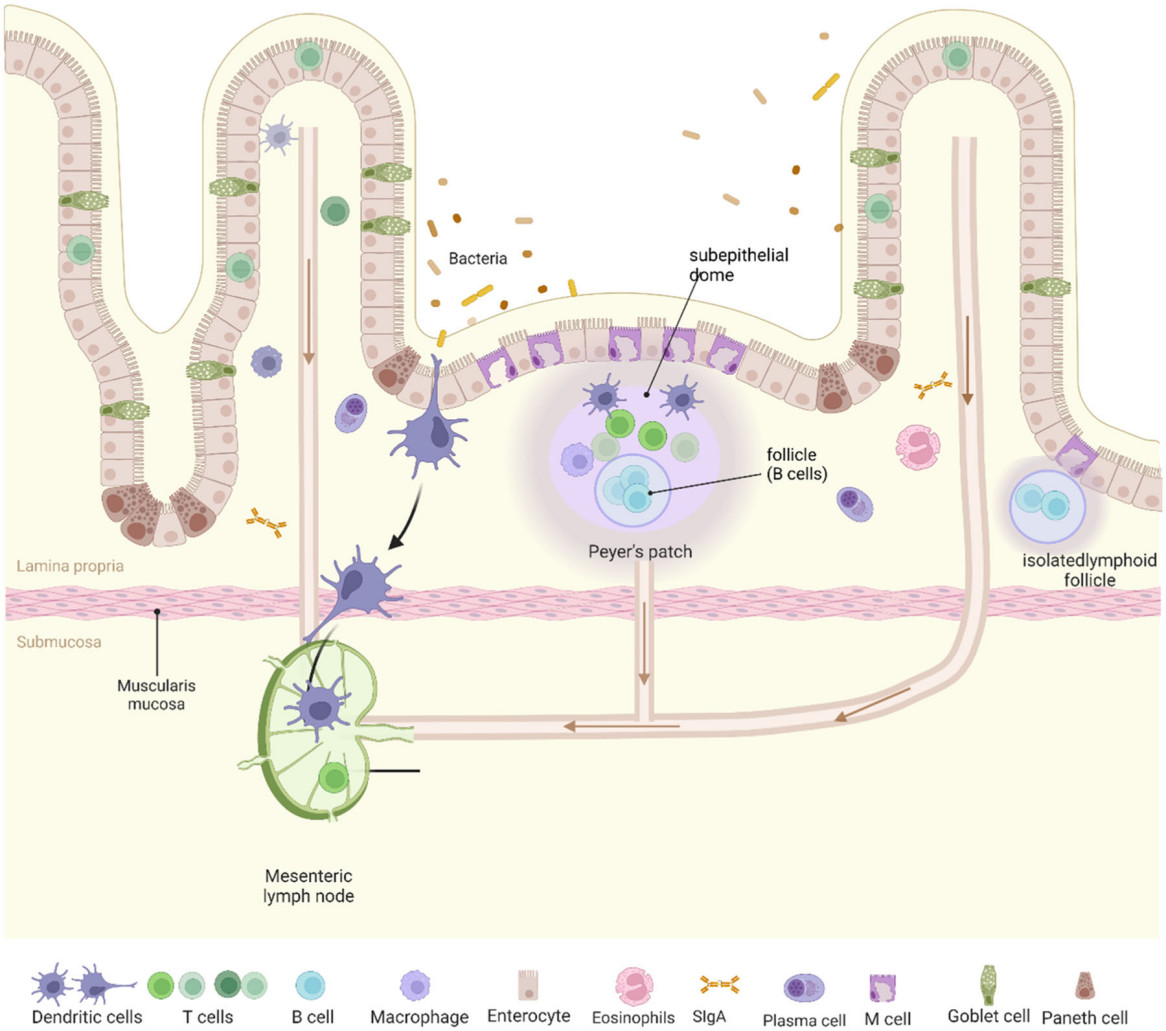
Composition of the intestine‐associated lymphoid tissue (GALT). The intestinal mucosa of the small intestine is composed of a layer of epithelial cells that digest food and absorb nutrients. The tissue beneath the epithelial cells is called the lamina propria. These tissues are located in the intestinal wall and and are protected from the contents of the intestinal lumen by the epithelium. The most prominent lymph nodes in the body are the MLN, which are connected to the PP and intestinal mucosa via the outflow lymphatics. Both PP and MLN contain T‐cell areas and B‐cell follicles, and the independent lymphoid follicles are mainly composed of B cells. Lymphocytes are scattered throughout the mucosal tissue and are mainly effector T cells and antibody‐secreting plasma cells. Effector lymphocytes are present in the interepithelial and lamina propria layers, and lymphatic fluid drains from the lamina propria to the MLN.

The GALT is a lymphoid tissue located in the submucosa of the intestine and comprises the Peyer's patches; isolated lymphoid follicles (ILF), which are found throughout the intestine; the esenteric lymph node (MLN); the vermiform appendix and diffuse immune cells.^18^ PP and ILF are connected to the MLN via lymphatic vessels, which are the sites of antigen recognition and activation of intestinal mucosal immune cells. A layer of follicle‐associated epithelium (FAE) separates the lymphoid tissues from the stomach lumen. It consist of mainly intestinal epithelial cells with scattered microfold cells (M cells).^20^ M cells are epithelial cells with specializations, which are cytosolic transporters of antigens. They absorb chemicals and particles from the gut lumen via endocytosis or phagocytosis and transport them via membrane‐bound vesicles to APC to initiate mucosal immune response—a process known as transcytosis^21^, ^22^, ^23^, ^24^ (Figure 1).

The majority of Intraepithelial lymphocytes (IELs) can be found in the small and large intestines. 90% of IELs are T cells, 80% of which are CD8+ T cells. They express chemokine receptor CCR9 and α_E_β_7_ integrin (CD103), allowing them to bind to CCI25 and E‐cadherin expressed by intestinal epithelial cells and localize to the inner intestinal epithelium. IELs are mainly divided into two categories: alEL and bIEL. alEL is primarily conventional CD8^+^CTL activated by an antigen, expressing αβTCR and CD8αβ heterodimer, with a TCR of limited multiplicity. It can be directly activated by specific MHC‐antigenic peptides to exert mucosal anti‐infection effects like the conventional class I MHC‐restricted cytotoxic T cells, killing virus‐infected cells.^25^ bIEL is intrinsically immune lymphocytes that migrate directly from the thymus, express αβTCR, γδTCR or CD8αα homodimer, and high levels of the C‐type lectin receptor NKG2D to promote barrier integrity and protective immunity.^26^, ^27^

By contrast, efficacy or memory CD4 T cells predominate in lamina propria lymphocytes (LPLs). More than 95% of intestinal lamina propria T cells bear the αβ isotype of the antigen‐specific T cell receptor. Recent studies show that the LP also carries a large population of heterogeneous innate lymphocytes (ILCs), including not just classical NK cells but also other cell types that can be categorized into three different groups—ILCs1, ILCs2, and ILCs3—based on cytokines and transcription factors they express. ILCs contribute to hosting defense against infection, metabolic homeostasis, and tissue repair.^28^

The overload of effector lymphocytes in the mucosal tissues, even in the absence of disease, may seem like a chronic inflammatory response and maintains the favorable relationship between the host and bacteria.

## 4 RESPONSES TO ENTERIC PATHOGENS: COMMUNICATION IS NECESSARY FOR THE NEIGHBORHOOD

Resisting infectious agents is the primary function of the mucosal immune response, thus the host must be able to create a variety of immune responses to meet the challenge posed by certain pathogens. The innate immune system is the initial line of defense against pathogens, which is a characteristic shared by all immune responses. Thus we only emphasize characteristics that the intestine monopolizes.

The most important mechanism of innate immunity involves the epithelial cells themselves. The intestinal mucosal layer of mucus, the glycocalyx on the microvilli of absorptive intestinal epithelial cells, and the tight cell junctions between intestinal epithelial cells all work together to produce a physical barrier that is normally impenetrable to intruders.^29^

On the apical and basal surfaces of epithelial cells are TLRs, the first pattern‐recognition receptors (PRRs) that detect pathogen‐associated molecular patterns (PAMPs).^30^ They are either situated on the cell membrane (TLR1, TLR2, TLR4, TLR5, TLR6, and TLR10) or inside vesicles (TLR3, TLR7, TLR8, TLR9, TLR11, and TLR13).^31^ Ligation of TLRs proteins stimulates cellular responses induced are mediated by several signaling molecules and cascades, incorporating the adapters MYD88 and TIR domain‐containing adapter protein inducing IFNβ (TRIF). This leads to the activation of mitogen‐activated protein kinase (MAPK) signaling and the transcription factors NF‐κB and interferon‐regulatory factor 3 (IRF3), followed by the overexpression of genes encoding pro‐inflammatory cytokines, such as IL‐1 and IL‐6, and those for chemokines including CXCL8, which is a potent neutrophil chemoattractant, and CCL2, CCL3, CCL4, and CCL5, which attract monocytes, eosinophils, and chemokine CCL20, which attracts immature DC cells to the epithelial surface.^31^, ^32^, ^33^

Epithelial cells also express the cytosolic NOD‐like receptors (NLRs) during enteric infections, which, according to their amino‐terminal domain, can be divided into three major subfamilies: the NOD proteins(NOD1, NOD2, and NLRC4), the NLRP family or the NAIPs.^34^ The NF‐B and MAPK signaling pathways are activated when NOD proteins bind to the receptor‐interacting serine/threonine kinase (RICK).^34^ By contrast, NLR proteins form inflammasome and activate caspase 1, which cleaves pro‐IL‐1 and pro‐IL‐18.^35^

Recent studies recently found that another important anti‐infection mechanism for epithelial cells is autophagy. In this process, a double‐membrane shard in the cytoplasm termed phagophore devours various cytoplasmic contents to make an autophagosome, which coalesces with lysosomes to degrade the contents. When autophagy is disrupted, bacteria penetrate the body, and NFκB‐mediated inflammation is activated. This process is promoted by NOD1 and NOD2.^36^, ^37^

**Figure 2:**
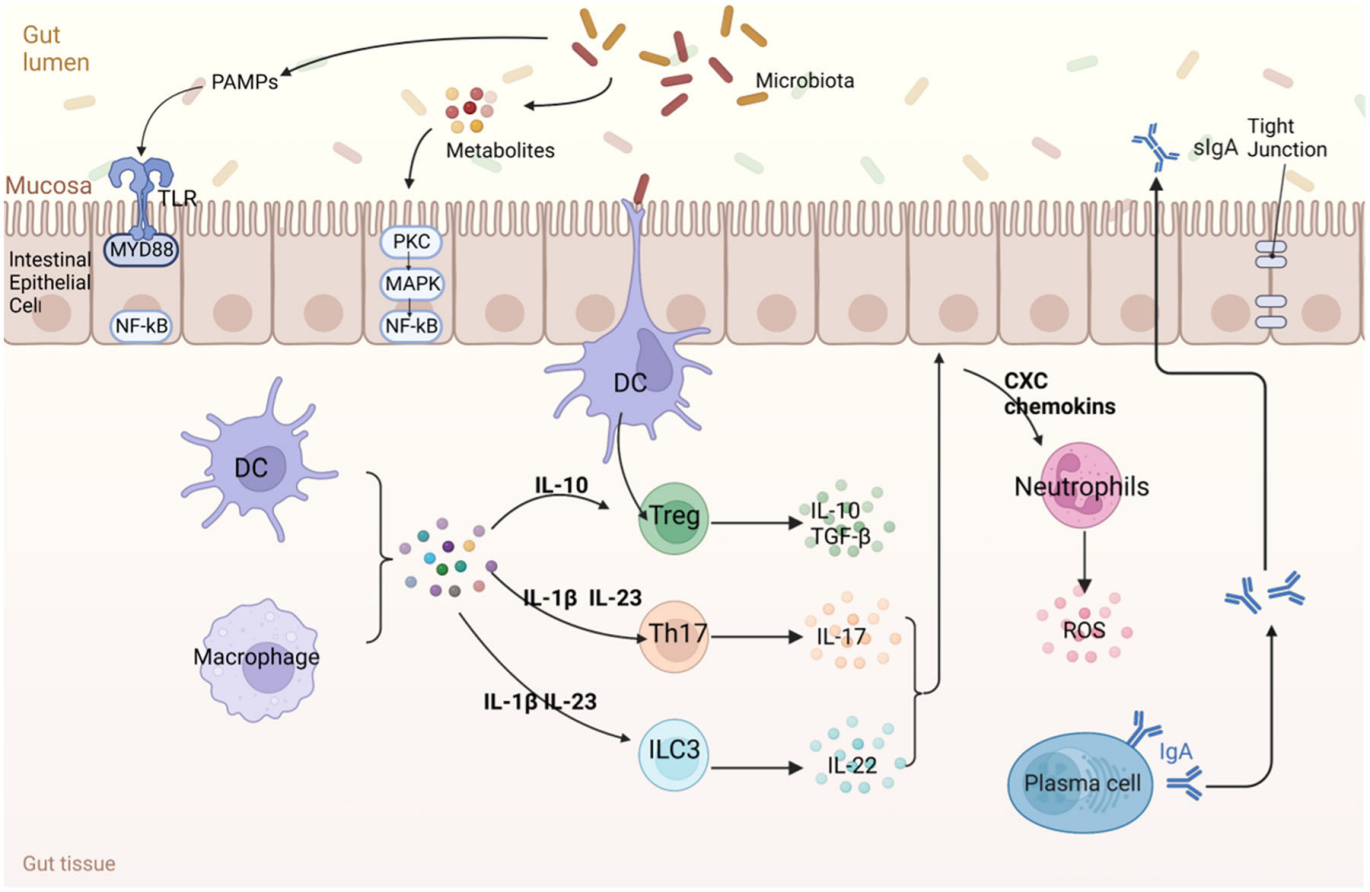
Mucosal immune response against pathogenic microbial infections. ① TLR from innate immunity recognizes dysregulated microbiota and leads to the initiation of NF‐κB‐dependent downstream spindle inflammation positive runners. The NF‐κB signaling cascade can also be activated by microbial derivatives. ② Gut bacteria can also transmit to the gut lumen and are sampled by DCs. ③ DCs create a tolerogenic response by stimulating Treg cells to secrete IL‐10, which in turn induces a tolerogenic response. Pathogens activate macrophages and DCs, which stimulates several subpopulations of T cells, including Th17and ILC3s, among others, secrete IL‐17 and IL‐22. ④ These cell subsets stimulate the intestinal epithelium to release neutrophil‐attracting CXC chemokines, hence enhancing the host response.⑤ Secreted lgA from plasma cells can also regulate microbiota and pathogens.

When innate defenses are broken down, adaptive immune responses are induced. If pathogen penetrates the subepithelial space, it may contact TLRs on the inflammatory cells there, thus sparking the cascade of inflammatory mediators and activation of local antigen‐presenting cells such as DCs. DCs will express costimulatory molecules and cytokines such as IL‐1, IL‐6, IL‐12, and IL‐23^38^ and facilitate the development of effector T cells, including TH17 cells, γδ T cells, NK cells, and ILC3s to secrete IL‐17 and IL‐22.^39^ These cells amplify the host immune response by stimulating the IEL to secrete CXC‐chemokines attracting neutrophils.^40^, ^41^ Antimicrobial peptides (AMPs) are produced in response to IL‐17 and IL‐22, and these AMPs alter the gastrointestinal tract's microbiota makeup.^42^ Similarly, IgA‐producing B lymphocytes are produced in PPs and MLN, generating plasma cells that accumulate in the lamina propria to regulate gut microbes and harmful bacteria by secretory IgA (sIgA).^43^ These antibodies mutually combine^44^ and regulate the microbiota make‐up of the digestive tract^45^ to control causes and effects of inflammation^46^ and defense against penetration pathogens^44^ (Figure 2).

## 5 MUCOSAL TOLERANCE: AWESOME HARMONY WE ESTABLISH

Antigens of commensal bacteria usually do not induce an inflammatory immune response. The mucosal immune system has evolved with these exogenous antigens and thus developed mechanisms to respond to innocuous antigenic substances, which is a very complicated process that involves triggering some immune responses while suppressing others.

There are shreds of evidence that the organized structures of the GALT, such as Peyer's PP and ILF, are crucial to the immune system's ability to recognize inhaled antigens on particles. The MLN has been identified as the site of oral tolerance induction because mice lacking CCR7, a critical chemokine receptor for cell migration to lymph nodes, were unable to generate oral tolerance.^47^ At the same time, tolerance can be developed orally without the presence of Peyer's patches.^48^, ^49^

It is becoming clear that tolerance requires CD103+ DCs to move from the lamina propria, where they collect antigen, to the MLN, where they stimulate the differentiation of naive T cells into Tregs via a TGF and retinoic acid‐dependent process.^50^, ^51^, ^52^, ^53^ After taking up the antigen, CD103+ DCs exhibit elevated RALDH2 levels, an enzyme metabolizing retinol into retinoic acid. Retinoic acid programs newly primed T cells to migrate to the MLN by means of gut‐homing markers, such as chemokine receptor CCR9 and the integrin α4β7.^54^ In addition, CD103+ DCs express significant quantities of indoleamine 2,3‐dioxygenase (IDO), an enzyme that catabolizes tryptophan and activates Foxp3+ Tregs.^55^ After the Tregs are generated in the MLN, they migrate to the lamina propria, where they are expanded to evoke tolerance.^56^

The two major aspects accounting for mucosal tolerance are clonal deletion, clonal anergy of antigen‐specific T cells, or the induction of active suppression mediated by Treg cells.^57^, ^58^, ^59^

**Figure 3:**
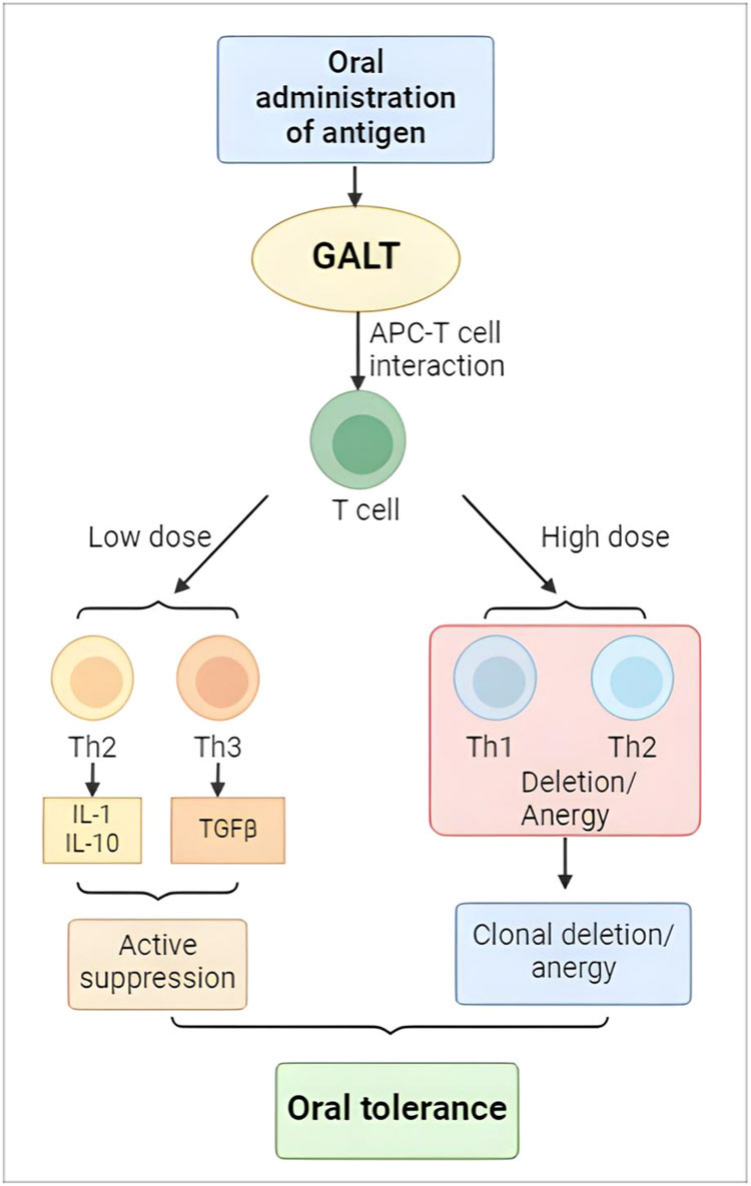
The proximity of feeding antigen determines the various oral tolerance processes.

Antigen dosage is the key determinant of which form of peripheral tolerance develops following oral administration of antigen.^60^ High antigen concentrations primarily induce anergy or clonal deletion, whereas low antigen levels promote active control of suppressor cells(Tregs).^61^ These two forms may occur simultaneously, but they are distinct (Figure 3).

The following criteria were used to distinguish between these two mechanisms: (i) investigations in which low dosages of an auto antigen suppress experimental autoimmune disorders by stimulating the production of regulatory cells that are suppressed through the secretion of inhibitory cytokines such as TGFβ^62^; (ii) investigations which showed after large doses of clonal antigen anergy happen, but there's with no evidence of active suppression^63^, ^64^; (iii)investigations revealed transferrable suppression following mucosal tolerance,^65^ including two aspects one that was abolished by treatment with low dose cyclo­phosphamide and one that was not. The difference was determined by the dose of the antigen^66^; (iv) investigations of a strong contrast demonstrating the direction the two distinct mechanisms depend on the dose.^67^ Low versus high dose feeding regimes elicit unique mechanisms of resistance to autoimmune uveitis, according to reports.^68^

Low antigen dosages stimulate antigen‐specific regulatory cells, which involve a presentation of antigen in the GI. Such presentation initially induces Tregs, which recognize antigens and subsequently secrete the suppressive cytokine TGFfJ. Th2 responses in the GI then promote the secretion of IL‐4 and IL‐IO. These antigen­specific Tregs move to lymphoid organs where they release cytokines that dampen an immune response to illness without targeting a specific antigen (bystander suppression).^68^ Several variables can affect the formation of regulatory T cells, such as requirements for costimulation, the cytokine environment in which the immune response is created, and the differential development of epitopes that may selectively activate certain regulatory cells are discussed.

**Figure 4:**
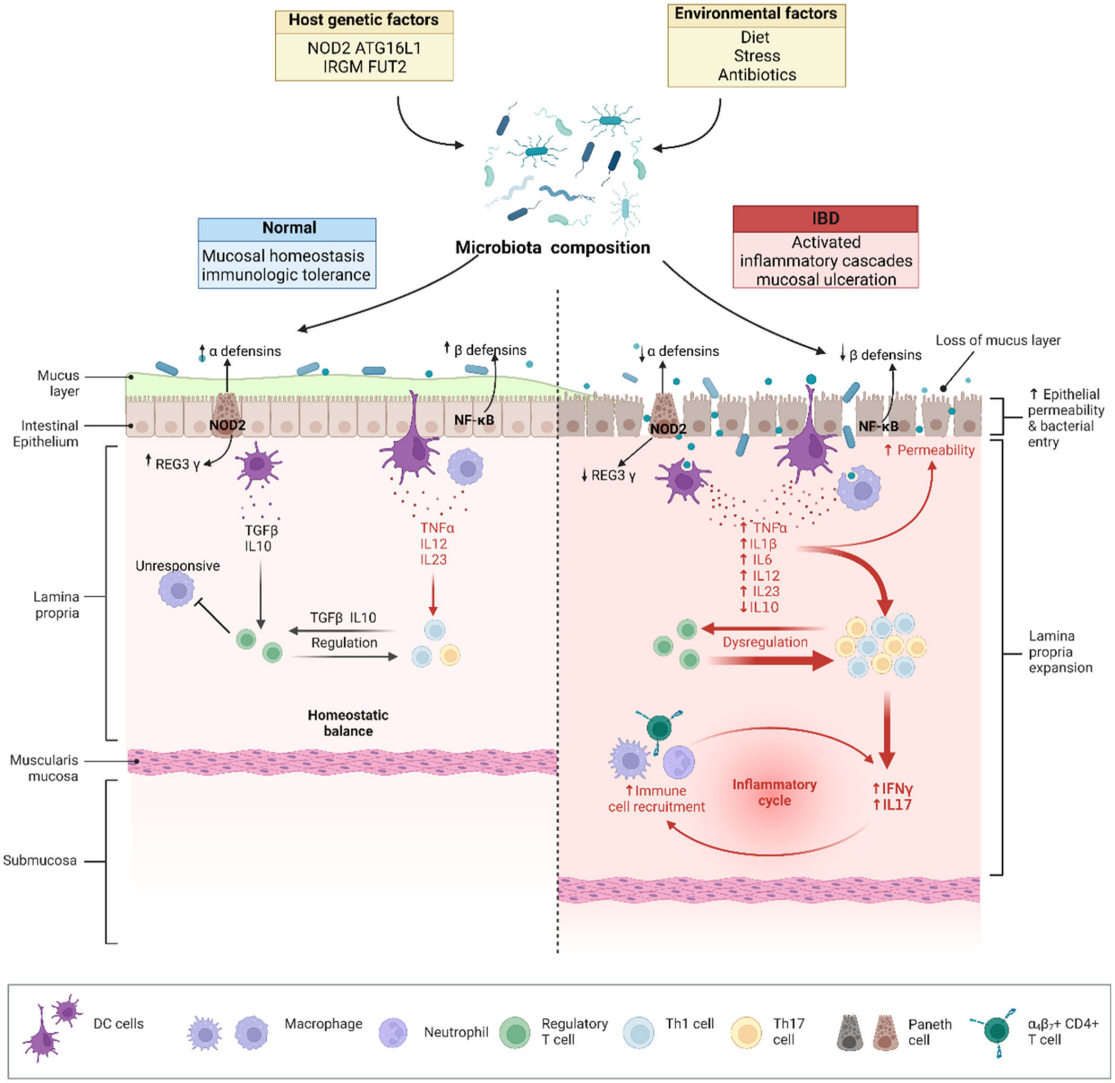
Immune response in IBD. Genetic and environmental variables affect the composition of the intestinal microbiota, which plays a protective role in normal hosts but contains an unbalanced ratio of helpful and aggressive bacterial species (dysbiosis) in inflammatory bowel disease (IBD). To put it simply, antimicrobial proteins, such as REG3γ, are induced under healthy conditions (left panel) and pathogens are kept in check as a result to maintain equilibrium. In IBD (right panel), hyperactivation of T Th1 and Th17 cells, increased permeability of tight junctions, decreased regulatory Treg cells, and decreased REG3 and IL‐10 are all results of chronic inflammation that is allowed to run amok.

High doses of antigen promote systemic antigen presentation after antigen transits the digestive tract and gets into the systemic circulation, which induces disability of Th1 cells, primarily via clonal anergy. Anergy is defined as a state of T cell unresponsiveness characterized by a lack of proliferation, IL‐2 production, and IL‐2 receptor (IL‐2R) expression.^69^ Experimentally, anergy can be distinguished from clonal deletion by confirming the existence of antigen‐specific TCR clonotypes or by releasing cells from their anergic state by preculturing them in IL‐2.^70^ The factors determining the degree of the clonal anergy following high doses of antigen are still unknown; nevertheless, the amount of antigen passing into the systemic or portal circulation or the filtration of the gut may be the most likely impact causes. It is unknown why high concentrations of antigen result in decreased active suppression, however it may be related to the activation of cells involved in the production of active suppression.

Once oral tolerance is induced, numerous antigen‐specific immune responses Th1 and Th2 are suppressed, including immunoglobulin (IgM, IgG) synthesis^71^, ^72^, ^73^ and creation and discharge of immunoregulatory cytokines, such as TGF‐β, IL‐10 and IL‐4.^61^, ^73^

A significant conceptual advance in the study of oral tolerance is the recognition of the importance of TGF‐β. It is an elementary link between different subspecies of induced Treg cells and thus is involved in the transformation of CD4 + CD25‐ cells into CD4 + CD25 + T cells via Foxp3.^74^ A membrane‐bound form of TGF‐β has also been identified, and CD4 + LAP + T cells are involved in suppression via mechanism depending on TGF‐β.^75^, ^76^, ^77^

Commensal bacteria are a potential threat, so unlike soluble food antigens, they do not induce a state of systemic immune unresponsiveness. When these pathogens infiltrate the circulation through the mucosa, they can trigger the normal main systemic immune response.

## 6 THE MODULATING FUNCTION OF MICROBIOTA: THE IMPACT THAT NEIGHBORS BRING TO US

Possibilities for microbiota to regulate the host's physiology include the development of an incredibly wide metabolite repertoire. Colonic microbial fermentation of undigested or partially digested dietary fibers produces short‐chain fatty acids (SCFAs) like butyric acid, propionic acid, and acetic acid, which are able to access through the intestinal epithelia and interact with host cells, thereby influencing immune.^78^ These microbiota‐generated metabolites are critical energy sources not only for the gut microbiota but also for intestinal epithelial cells (IECs) and have a variety of regulatory roles in host physiology and immunity.^79^ SCFAs have numerous impacts, including improved epithelial barrier function and immunological tolerance, which promote gut homeostasis via distinct mechanisms: increased mucus production by intestinal goblet cells^80^; inhibition of nuclear factor‐κB (NF‐κB)^81^, ^82^; activation of inflammasomes and subsequent production of interleukin‐18 (IL‐18)^83^; increased secretion of secretory IgA (sIgA) by B cells^84^; decreased expression of T cell‐activating chemicals on APCs, such as dendritic cells (DCs)^85^ and macrophage^86^; and elevated levels of regulating T (Treg) cells in the colon, including their expression of forkhead box P3 (FOXP3)^87^, ^88^ and their production of anti‐inflammatory cytokines such as IL‐10.^89^

Another mechanism by which the microbiota affects the host is by triggering epigenetic changes in host cells. To achieve this, SCFAs and other microbial metabolites regulate histone acetylation. The modulation of Treg differentiation by butyrate is a powerful example of microbiota influence on the immune system via epigenetic control.^90^ The microbiota also causes alterations in DNA or histone methylation.^91^, ^92^

Recently, researchers discovered bacterial extracellular vesicles (EVs) that elicit distinct host responses based on the specific microorganism they come from.^93^ Similar to LPS, the immunogenicity of microbiota‐derived extracellular vesicles (EVs) varies depending on the type of microorganisms. EVs from pathogenic bacteria are linked to pro‐inflammatory responses, while EVs from commensal bacteria trigger anti‐inflammatory responses. For instance, electric vehicles (EVs) derived from a symbiotic strain of *E. coli* bacteria increased the production of mucosal cytokines that are crucial for the host's immune response.^94^ Although our understanding of microbiota‐derived extracellular vesicles (EVs) primarily comes from studying their impact on the stomach, recent research has revealed that these EVs also play a role in regulating the immune system throughout the body. Specifically, microbiota‐derived EVs prepare neutrophils to respond to secondary stimuli by triggering inflammatory reactions.^95^

### 6.1 Regulation of epithelial cells by the microbiota

To limit inflammation and microbial translocation, the primary strategy taken by the host is reducing microbe interaction with the epithelial cell surface, which is achieved through the cooperative action of epithelial cells, mucus, IgA, antimicrobial peptides and immune cells.^96^ Antimicrobial peptides produced by intestinal epithelial cells exert antimicrobial effects, including an enzymatic attack on the bacterial cell wall and the rupture of the bacterial inner membrane.^97^ One of the most characteristic mucosal antimicrobial peptides is RegIIIγ, the production of which in a MyD88‐dependent way is carefully regulated by the flora.^98^ Indirectly, microbiota affects intestinal epithelial cells through cytokine generation by innate and adaptive immune cells in response to microbial colonization.^99^

### 6.2 Regulation of innate immunocytes by the microbiota

Antigen‐presenting cells are regulated by microorganisms. The ubiquitous bacterial fermentation products SCFA is capable of activating G protein‐coupled receptors on epithelial and hematopoietic cells and inhibiting histone deacetylase (HDACs).^83^ Elevation of circulating SCFA led to the generation of macrophage and dendritic cell (DC) precursors, followed by DCs with a high phagocytic capacity populating the lungs.^100^ SCFA can also change macrophages in the area by influencing their gene expression profile.^86^ In addition, microbiota also controls the homeostatic replenishment of monocyte‐derived macrophages in the intestinal mucosa.^101^

Through TLR signaling, microbial molecules such as lipopolysaccharides maintained steady‐state production of neutrophils and primed neutrophils against bacterial infections.^102^ The microbiota also affects the number of innate cells like basophils that circulate through the bloodstream.^103^ On the other hand, stimulation of neutrophil function via microbiota proves to promote neutrophil “aging” through tonic sensing of TLR ligands.^104^

### 6.3 Regulation of nonclassical lymphocytes by the microbiota

It has been demonstrated that nonclassical lymphocytes, such as ILC, MAITs, NKT, and NK cells, are enriched at barrier sites during early life and can coordinate the interaction between the host and its microbiota.

ILC can produce IL‐22, which can help keep some of the microbiota in the mucosal lymphoid structures, like Alcaligenes bacteria, under control.^105^ Recent evidence supports the idea that defined commensals may preferentially impact ILC3, the most noteworthy subset of ILC, but the mechanism remains unclear. As an illustration, SFB colonization can increase ILC3's ability to produce IL‐22 in an IL‐23‐dependent manner.^106^

γδT cells can be activated by TCR‐mediated responses as well as responses to cytokines such as IL‐1 and IL‐23,^107^, ^108^ both of which can be promoted by the microbiota's action on tissues. Maintaining the homeostasis of liver‐resident T‐17 cells via lipid antigen/CD1d‐dependent pathways, microbiota colonization stimulates the generation of T cells at barrier sites in an IL‐1 or IL23‐dependent manner.^109^, ^110^, ^111^

Nonclassical MHC molecules can present microbiota antigens with particular chemical modifications or amino acid motifs, thus making them possible to recognize microbiota‐derived antigens or metabolites. In evolutionary terms, MAIT cells are limited by the non‐polymorphic and highly conserved MHC‐Ib molecule, MHC class I‐related protein 1 (MR1),^112^ and are highly responsive to bacterial owing to their capacity to identify metabolic intermediates of the microbial riboflavin synthesis pathway.^113^ In addition, the growth of MAIT cells depends on the microbiota, as they are absent in aseptic mice.^114^

When CD1d, an MHC class I‐like molecule, is present, NKT cells respond to lipid antigens from both host cells and microbes. The accumulation of NKT cells in the colonic lamina propria is restricted by commensal colonization in early life.^91^

### 6.4 Regulation of T cell response by the microbiota

CD4 + T cells, most of which are effector or memory T cells in the intestinal LP, respond greatly differently based on the different niches of colonization, antigen type, and metabolic property of gut microbiota. Upon activation by microbiota and presentation of antigens by APCs, CD4 + T cells generate distinct subsets and the functional plasticity of specific T cell subsets,^115^ among which are Tregs and various T helpers (Th1) cells such as IFN‐γ, IL‐4, B cell regulating, and IL‐17 producing Th1, Th2, Tfh, and Th17 cells.^116^, ^117^ SCFAs from the gut microbiota have been shown to modulate T cell differentiation depending on their concentration and the surrounding immune environment.^118^

Th17 is crucial for host defense against external infections because of its ability to make IL‐17. Th17 cell frequencies within the GALT of germ‐free mice are significantly decreased.^119^ The colonization of commensal microbiota, particularly Clostridia‐related segmented filamentous bacteria(SFB), has been shown to strongly stimulate the formation of Th17 cells in the small intestine.^120^ SFB promotes CD4 + T cell differentiation into RORγt‐expressing Th17 cells via inducing the expression and release of serum amyloid A (SAA) from intestinal epithelial cells (IECs), which stimulates IL‐22 production by increasing IL‐1 and IL‐23 in CX3CR1+ phagocytes. RORgt+ CD4 + T cells' IL‐17 production can be upregulated by IL‐22 because of its ability to boost SAA‐mediated IL‐1 production by phagocytes.^106^, ^121^

The makeup of intestinal microbiota has a major impact on the repertoire of regulatory T cells (Tregs) that express the forkhead box P3 transcription factor (Foxp3‐).^122^ Symbiotic bacteria, such as Clostridia strains cocktail and *Bacteroides fragilis*, were identified to possess Treg‐inducing activity as well as the production of IL‐10.^123^ In addition, SCFAs, particularly butyrate, have demonstrated to influence the formation and activity of regulatory T cells in the colon via enhancing acetylation of the Foxp3 locus in Tregs^90^, ^124^ and promoting TGFβ production, which indirectly contributes to the development of colonic Tregs.^121^ Moreover, the expression of transcription factor RORγt+ of RORγt+ Tregs, which are a separate group of Tregs in the colon, is induced by gut microbiota.^125^

Germinal centers (GCs) have been found to be the sites where high‐affinity antibodies are produced, and Tfh has been found to be the primary cell subpopulation responsible for controlling B cells in these GCs. Transforming growth factor development is impaired in GF animals, however using a Toll‐like receptor 2 (TLR2) agonist to activate intrinsic MyD88 signaling rescues this defect.^126^ Extracellular ATP (eATP), an important signaling molecule derive from microbiota, can restrict Tfh cell formation and GC reaction in the PPs by means of P2X7, regulate Tfh cell abundance, and influence the high‐affinity sIgA response against intestinal colonization bacteria, which leads to enteropathogenic infection.^127^, ^128^

### 6.5 Regulation of B cell immune response by the microbiota

IgA is the most abundant isotype of antibodies and is essential in promoting early interactions with the microbiota and preserving microbial diversity and compartmentalization.^129^ The production of intestinal IgA is greatly influenced by the microbiota because, physically, they are very close.^130^ It is possible for secretory IgA to be generated in either a T‐independent (TI‐IgA) or T‐cell‐dependent (TD‐IgA) fashion (referred to as ID‐IgA).

A fraction of antimicrobial polyreactive IgA in the gastrointestinal tract is produced via the high‐capacity, low‐affinity T‐cell‐independent passage.^131^, ^132^ Lack of intestinal microbial stimulation would probably impair the formation of distinct lymphoid tissues, which is a major generation site for TI‐IgA and consequently leads to a decrease in the number of IgA+ plasma cells and reduces the abundance of IgA.^133^, ^134^, ^135^ SFB potently promotes TI‐IgA production by stimulating the postnatal development of isolated lymphoid tissues and tertiary lymphoid tissue in the gastrointestinal tract.^130^, ^131^

Nevertheless, the majority of intestinal IgA, especially that directed against bacterial protein antigens, is T‐cell reliant and a component of a low‐capacity, high‐affinity route.^131^, ^132^ For PP subepithelial B cells to produce TD‐IgA, affinity mature, and undergo class switch recombination, they must engage with antigen‐loaded dendritic cells in a CCR6‐dependent way.^44^, ^136^ Bacteria such as SFB and Mucispirillum sp. can induce the production of TD‐IgA, which may be due to enhancing acquisition of their antigens by DCs and supporting the growth of T helper 17 and T helper 1 cells in the gut.^132^

It is not just intestinal IgA that binds gut bacteria, but also certain subclasses of IgG and IgE, most of which are triggered via a T‐cell‐independent pathway.^137^ Wide range of microbes encounter at mucosal sites downregulates IgE to baseline levels while triggering the IgA isotype switch. IgE production is influenced by the gut microbiota via a pathway opposite to gut microbiota‐regulated IgA response. Without microbial exposure, active CD4 + T cell‐dependent B cell isotype switches to IgE at PPs, thus producing high levels of IgE.^138^ IgG that may identify bacterial surface antigens, such as murein lipoprotein (MLP), which are expressed on certain Gram‐negative pathogens, can be decreased under homeostatic settings by selective Gram‐negative gut symbionts.^121^

## 7 DISEASE AND TREATMENTS: NEIGHBORHOOD CONFLICTS ERUPT FROM TIME TO TIME

Under normal circumstances, the host and its microbiota endure mutualistic partnerships to achieve and maintain homeostasis. Nevertheless, when the dynamic crosstalk between the two goes amiss due to disorders of the microbiota or the host, dysbiosis will ensue. Studies have confirmed the role of an altered microbiome in human disorders and diseases (Table 2).

**Table 2:** Microbial metabolites or components that are implicated in disease.

Human disease and preclinical models	Microbial metabolites or components	Refs
Inflammatory bowel disease	SCFAs	[139]
	B vitamins	[89]
	Compound K	[89]
	AHR ligands	[140]
Colorectal cancer	SCFAs	[89, 141]
	B vitamins	[89]
	N1,N12‐diacetylspermine	[141, 142]
Bacterial vaginosis and other sexually transmitted infections	Polyamines	[143]
Obesity and metabolic syndrome	TAMO	[144]
	SCFAs	[145]
Infectious colitis (Clostridium difficile)	Bile acids	[146]

### 7.1 Inflammatory bowel disease (IBD)

Immune recognition of gut microbiota triggers multiple inflammatory disorders, among which IBD is the most representative and well‐researched. IBD encompasses primarily ulcerative colitis (UC), which is characterized by bloody mucous diarrhea, and Crohn's disease (CD), the hallmark of which is abdominal pain.^147^ Several laboratory and clinical studies have demonstrated that gut microbiota is a major driver of pathogenic inflammation.(Figure 4).

The intestinal mucus layer shields the host epithelium from intestinal contents as the first line of defense. The mucus layer contains bacteria and dietary proteins, peptides, and IBD is characterized by a breakdown in the intestinal mucosal barrier.^148^, ^149^ Recent research lends credence to the idea that a weakened colonic mucus barrier contributes to the etiology of UC. Specifically, a significant decrease in the structural MUC2 protein was detected in the colonic mucus of UC patients.^150^ Moreover, in ulcerative colitis, a particular malfunction of the intestinal mucus layer has been noted. Defective glycosylation that allows bacteria to penetrate the otherwise impenetrable inner colonic mucus layer has been observed in UC patients and mouse models.^151^ An intricate protein complex, TJs, which regulates the paracellular gap and permeability, is responsible for determining the paracellular barrier function of the intestinal epithelium. In IBD intestinal tract, the continuous, linear chain pattern typical of TJs in epithelia changes to a granular appearance with strand breaks and loss of continuity, and fewer strands are horizontally aligned.^152^, ^153^

Panniculocyte defensins and Panniculocyte alterations also contribute significantly to Crohn's disease. A study found that Paneth cells from IBD patients overexpress NOD2.^154^ Expression of alpha‐defensin HD5 and HD6 mRNAs and proteins reduced in the small intestine of Paneth cells from UC patients. Paneth cells, which are usually rare in the colon, may be found in the inflamed IBD colon, expressing α‐defensins HD5 and HD6 as well as lysozyme and sPLA2.^155^, ^156^ In contrast, Low PPAR‐regulated HBD1 and impaired HBD2 and HBD3 induction characterize CD.^157^, ^158^

Clinical observations can indirectly demonstrate the major role of microbial communities. For example, in humans, the first evidence implicating gut microbiota in the etiology of IBD is that CD can be improved by surgical repair to redirect fecal flow^159^ and increasing mucosal‐associated bacteria in the nonterminal ileum after ileocecal resection for CD may trigger postoperative recurrence in patients.^160^ Furthermore, antibiotics, such as metronidazole, ciprofloxacin, or rifaximin, advantage in the management of inflammatory bowel disease.^161^, ^162^ Due to these intricate and strong arguments, there is currently little doubt regarding the involvement of bacteria in disease initiation.

Metabolomic and metagenomics investigations have shown that active IBD patients have a notable imbalance in their microbial composition. There is a reduction in the population of helpful bacteria, specifically those belonging to the groups Bacteroidetes, Firmicutes, and Lachnospiraceae (Clostridia cluster IV and XIVa). Additional research has also demonstrated a reduction in the presence of the bacteria species Roseburia hominis and Faecalibacterium prausnitzii, which are known to produce butyrate.^163^ Faecalibacterium species have the ability to promote the production of anti‐inflammatory cytokines, including IL‐10, which leads to a reduction in pro‐inflammatory cytokines in patients with inflammatory bowel disease (IBD).^164^ UC and ileal CD have been associated with a reduction in Faecalibacterium prausnitzii. Notably, the normalization of F. prausnitzii happened after UC patients achieved clinical remission.^165^ Research has also shown a substantial rise in the prevalence of detrimental Firmicutes species, Fusbacterium species, and Proteobacteria strains among patients with inflammatory bowel disease (IBD). The presence of adherent and invasive bacteria belonging to the facultative anaerobes genus Enterobacteriaceae, such as adherent‐invasive *Escherichia coli* and other Fusobacterium species, is much higher in individuals with ileal CD and UC.^166^

Furthermore, there is growing data indicating that viruses can stimulate both the innate and adaptive immune responses at the epithelial gut barrier, alongside bacteria. This viral activity also plays a role in the development of inflammatory bowel disease (IBD). Other studies have shown that there is an increase in the abundance of Caudovirales phages, Anellovirus, enteroviruses, Norovirus, and other eukaryotic viruses in CD and UC.^167^ Caudovirales (tailed bacteriophages) are the most studied.^167^, ^168^, ^169^ These phages establish communication with immune cells by infecting the bacteria and triggering the production of IFNs and Th1 cell‐mediated responses. IFNs are a class of cytokines that engage with the antiviral genes of the host. Cytokines have the ability to decrease the amount of virus present and regulate the reactions of macrophages.^170^ This connection creates a beneficial symbiotic environment for the virus to thrive within the host.

The new SARS‐CoV2 virus, which causes COVID‐19 infection, has the ability to trigger significant inflammation in the lungs and lead to gastrointestinal symptoms.^171^, ^172^ COVID‐19 receptor ACE2 expression is elevated in the intestinal epithelial cells of both UC and CD patients, regardless of the intensity of inflammation, in the terminal ileum and colon, as compared to control. Additional research is required to elucidate the mechanisms by which SARS‐CoV2 specifically affects the immunological pathways and impacts the progression and treatment of inflammatory bowel disease (IBD) in individuals.^173^

### 7.2 Colorectal cancer (CRC)

Colorectal cancer (CRC) is one of the most prevalent tumorswith established etiology of molecular, genetic, and environmental mechanisms. It globally accounts for about 10% and has the third highest incidence and mortality rate of all cancers.^174^ There is emerging evidence that pathogenesis of CRC is strongly influenced by microorganisms in the surrounding environment.

Dysbiosis, which represents a compositional change of resident gut microbiota, is typical in both fecal and mucosal samples of CRC patients. Metagenomic studies suggested that the abundance of Fusobacterium, Porphyromonas, Peptostreptococcus, and Prevotella, as well as altered fungi (mycobiota), has increased.^175^ Metagenomic and metabolomic analyses have shown consistent associations of the onset and progression of CRC with specific bacteria, such as Fusobacterium nucleatum, and several microbial metabolites.^176^, ^177^ Of particular interest to propose is the role of uncommon fungi, viruses, and archaea in colon cancer. The virome changes in patients with CRC: Betabaculus virus, Epsilon15likevirus, Mulikevirus, and Punalikevirus increased abundance, associated with increased severity and mortality. Colon eukaryotic viruses are capable of altering immunological homeostasis and inducing DNA changes via viral‐dependent pathways.^178^ It was observed that Malasseziomycetes enriched and Saccharomycetes lessened, and disturbance in the distribution of certain strains such as Aspergillus and Malassezia in CRC patients.^179^ It has also been reported that in mice, antifungal treatment aggravated CRC.^180^ Research on the relationship between archaea and CRC is still in its infancy, but it has been shown that CRC patients have more salt‐loving and less methanogenic archaea.^181^

Colorectal carcinogenesis is an intricate process. The following three mechanisms are most closely associated with mucosal immunity.

(1) CRC has the traits of long‐standing chronic inflammation in the mucosa, which is also a distinctive feature of IBD; thus, the immunological factors affecting IBD enteropathy described above can also affect CRC.
(2) The NF‐κB pathway in the mucosal epithelial cell is essential in controlling inflammation in cancer. Target genes of the NF‐κ B pathway encode pro‐inflammatory cytokines (e.g., TNF‐α, IL‐1, and IL‐6), chemokines (IL‐8), and enzymes (COX‐2) that are associated with tumor progression, survival, proliferation, and invasion.^182^ TNF‐α promotes tumorigenesis by inducing reactive oxygen species (ROS) production and promoting DNA damage.^183^ COX‐2 and IL‐8 can stimulate tumor progression and invasion by promoting angiogenesis.^184^
(3) The growth and spread of CRC is aided by Th17 cells and IL17. Laboratory detection of human CRC specimens found most colorectal tumors contain Th17 cells.^185^ In addition, altered expression profiles of genes in Th17 cells lead to a shorter disease‐free survival time in CRC patients.^186^
  Colorectal tumor stroma showed elevated levels of IL17A, which induces stromal production of factors that maintain tumor cell proliferation, survival, and angiogenesis.^187^
(4) Recently the role of innate systems, particularly innate lymphocytes (ILC), has been highlighted the in the pathogenesis of CRC. Intestinal ILCs drive both pro‐ and antitumor actions, tilting the scales in favor of tumor formation. The activation of ILC3 and the subsequent generation of cytokines appear to be crucial to their effect on colorectal cancer. The expression of IL‐23 is higher in human colon cancers than in healthy tissue, and it is associated with a worse prognosis and more aggressive disease.^188^ There are preventive and pathogenic functions for the ILC3‐driven IL‐22 pathway in colitis and cancer, respectively. By activating a DNA damage response pathway, IL‐22 shields the gut from the effects of genotoxic stress. When enterocytes are exposed to carcinogens, the loss of IL‐22 or IL‐22 receptors on epithelial cells causes a delay in tissue healing, increased inflammation, and tumor growth.^189^, ^190^

### 7.3 Mucosal vaccine

Vaccines could have a significant bearing on preventing the spread of disease even beyond the individual who receives them on the premise of extensive coverage.^191^ However, conventional vaccinations are given via systemic means, meaning they are injected with a syringe and needle. This approach typically results in minimal or nonexistent antigen‐specific immune responses at mucosal surfaces.^192^ Conversely, vaccination of the mucosal can generate both systemic immune protection (via antibody formation and immune cell‐mediated responses) and pathogen‐specific mucosal immunity provided the vaccine is administered via the right vehicle or is co‐administered with an adjuvant.^193^, ^194^, ^195^ Mucosal vaccines are preferable to injectable vaccinations not only because they are more effective immunologically, but also because they have a number of advantages from a production and regulatory perspective: (1) can be self‐administered, not requiring trained health professionals for administration and greatly decreasing the cost of mass immunization；(2) eliminating needle‐associated risks and cause less physical and psychological discomfort; (3) not following strict sterile procedures thus simplifying production and storage.^196^

We have a range of licensed vaccines proving the viability of oral immunization.^197^ It has been demonstrated that oral vaccines can generate mucosal IgA and serum IgG, as well as memory T cells and synergistic effectors in the gastrointestinal tract and salivary and mammary glands.^198^ Currently licensed vaccines include the oral polio vaccine (OPV), which was the first successful mucosal vaccine developed; live oral typhoid vaccine (Ty21a); cholera vaccine; rotavirus vaccine; and oral adenovirus vaccine.^199^

Several bacterial shuttle vector systems have then been tested for use as mucosal vectors. From 2015 to the present, bacteria used as mucosal vects include Salmonella spp., Listeria spp., Bacillus spp. Lactococcus and other novel bacterial vectors. Listeria and Salmonella have been extensively employed as vaccine vectors in several diseases, including cancer immunotherapy.^200^ However, It is important to consider the safety concerns associated with using live‐attenuated pathogenic bacteria as a recombinant delivery method. These bacteria have the potential to revert back to their pathogenic state, thus more research is needed.^201^ Consequently, researchers have investigated commensal bacteria to enhance safety without compromising effectiveness or altering the immunological response they elicit.

Some of these commensal bacteria tested include Bacillus spores, such as Bacillus subtilis (B. subtilis). These microorganisms have been utilized as a live vaccine vector system because of their safety, vitality, secretory capacity, and probiotic traits. B. subtilis has been employed in numerous research investigations as a vehicle for vaccines targeting viruses, harmful bacteria, and parasites in animal models.^202^ In addition, Oh and co‐workers^203^ used B. subtilis spores to express the protective antigen (PA) derived from Bacillus anthracis and evaluating the effectiveness of the construct in inducing an immune response in mice through various administration routes. Their research found that regardless of how the medication was given, the mice showed higher levels of active antibody titer, isotype profiles, toxin‐neutralizing antibody in their blood, and IgA in their saliva.

Lactobacilli, a potential commensal candidate, is being produced with a strong tolerance to acid and bile stress. It exhibits survival times exceeding 7 days. Lactobacilli have recently been proposed as a potential enhancer for several vaccine designs, showing the ability to modify both the innate and adaptive immune responses in clinical trials, particularly in gastroenterological conditions including rotavirus, cholera, and Salmonella infection,^204^ and additionally for respiratory infections such as Influenza, SARS‐CoV‐1, pneumonia, and Bacillus anthracis and so on.^204^

The components and metabolites of microbiota may impact vaccine effectiveness and adjuvant action. The gut microbiota of people from industrialized and poor nations is distinguished, and studies on vaccine efficacy vary among regions.^205^ People living in underdeveloped countries with poor sanitation have weakened immune responses to oral vaccines such as rotavirus,^206^ poliovirus,^207^ and cholera vaccines^208^ in Nicaragua.^209^ Even in the same country, Indian children in poorer regions showed reduced mucosal immune responses after vaccination with monovalent and trivalent oral poliovirus vaccine.^210^ As a matter of fact, an abundance of stool Actinobacteria was shown to be positively correlated with higher responses to oral and parenteral vaccines; nevertheless, Clostridiales, Enterobacteriales, and Pseudomonadales were related to lower vaccine responses.^211^ Animal studies provide further evidence that microbiota make up of one's digestive tract affects the efficacy of vaccines via mediating antibody responses. Mice treated with clarithromycin or doxycycline decreased in the production of induced antibodies after rejecting the hepatitis B virus surface antigen (HBsAg) vaccine. At the same time, enhanced immune responses to live attenuated Salmonella enterica serovar Typhi (Ty21a) vaccine.^212^ Furthermore, research proves that Correlations between Oscillospira and Streptococcus and the induction and protection of vaccine‐specific IgG and IgA were positive and negative, respectively.^213^

It is impossible to augment and direct a highly vaccine‐specific adaptive immune response without the use of mucosal adjuvants. Due to the powerful immunostimulatory capacity of bacterial‐derived components, they are a major source of possible adjuvants. For example, lipopolysaccharide (LPS), peptidoglycan (PGN), CpG DNA, and trehalose dimycolate (TDM) can strengthen the immune response against antigens through the activating TLRs, NLRs, and CLRs (C‐type lectin receptors).^214^ Mice lacking the TLR5, the specific receptor specific for bacterial flagellin, generated substantially fewer antibodies after receiving the trivalent inactivated vaccine (TIV) than normal mice. This result manifests that flagellin from the gut microbiota can act as an adjuvant to increase antigen responses to the TIV vaccine.^215^

## 8 CONCLUSIONS

A sophisticated system that is essential to maintaining good health is the mucosal immune system. The gastrointestinal microbiome, the largest symbiotic ecosystem with the host, is an essential contributor to maintaining intestinal homeostasis. The intestinal innate and adaptive immunity tolerate symbiotic microbiota and retain the ability to exert a pro‐inflammatory response towards invasive pathogens. When this balanced relationship is disrupted, ecological dysregulation and intestinal immune abnormalities develop, leading to microbiota dysbiosis, compromised integrity of the intestinal barrier, and pro‐inflammatory immune responses towards symbionts. Recent data has shown the fundamental relationship between the gut microbial environment and gastrointestinal disorders, and this presents a huge therapeutic opportunity for health promotion and disease management.

Recent accumulating evidence has revealed complex interactions between the gut microbiota and host cells in the gut ecosystem; nevertheless, there are still unresolved matters about undiscovered bioactive microbial metabolites and the interplay of gut microbes, encompassing bacteria, fungi, viruses, and other microorganisms. Advanced techniques such as metagenomics, meta‐transcriptomics, and metabolomics will provide new information about the functional properties of the microbiome and host cells in both healthy and diseased states. These strategies will enhance our comprehension of the internal mechanisms of the gut ecosystem and the various constituent elements involved. These potent instruments have been discerning the connection between gut microbial metabolites and systemic ailments, such as neurological disorders and cardiovascular conditions. Additional research involving thorough analysis of human samples, biological tests, and animal experiments.

## AUTHOR CONTRIBUTIONS

Keixin Tian contributed the central idea and wrote the initial draft of the paper. The remaining authors contributed to refining the ideas and finalizing this paper.

## CONFLICT OF INTEREST STATEMENT

The authors declare that they have no known competing financial interests or personal relationships that could have appeared to influence the work reported in this paper.

## ETHICS STATEMENT

Ethics approval and consent to participate is not applicable to this article.

## Supporting information

Supporting information.

## Data Availability

Data availability is not applicable to this article as no new data were created or analyzed in this study.
